# Practical Notes on Diagnosis and Treatment

**Published:** 1906-12-22

**Authors:** 


### Practical Notes on Diagnosis and Treatment.

**Salicylic Intoxication in Children.**

Salicylate of Sodium occasionally produces in young children a very alarming condition of pallor, delirium, and dryness of the tongue. Five grains three or four times a day is a full dose for a child of five or six years.—*Dr. Goodhart.*

**Catheters and Urethral Spasm.**

The obstacle presented to the passage of a catheter by *spasm* in the urethra may often be entirely removed by engaging the patient in conversation, and so directing his attention away from the part affected.—*Sir Wm. H. Bennett.*

**Strychnine in Pneumonia.**

As a cardiac stimulant in pneumonia strychnine should be given in full doses—say $\frac{1}{30}$ to $\frac{1}{20}$ grain—and these or smaller doses repeated every half hour until the heart becomes stronger or toxic symptoms begin to appear. The approach of the latter may be appreciated by observing a heightened character of the tendon jerks.

**Examination of the Pulmonary Apex.**

In one sense the upper axilla is a site of election in examination of the apex of the lung. We are here at a maximum distance from the trachea, and safe from the disturbing influence of any tracheal conduction. Any tubular breathing in the upper axilla means consolidation; any cavernous breath-sound means excavation. To make the examination effectual, the patient must place the palm of his hand on his occiput. This enables the stethoscope to reach the second rib between the muscular folds of the armpit.—*Dr. Wm. Ewart.*

**Mitral Stenosis.**

This condition is never congenital in the sense of a malformation, though in rare cases it may be a result of congenital endocarditis. It has been observed in very few instances under the age of five years.—*Dr. Sansom.*

**Hæmorrhage from the Bladder.**

In this condition, never pass a sound or catheter into the bladder whilst the bleeding continues, unless circumstances render such procedure absolutely necessary. The liquid extract of ergot in full doses will usually check hæmorrhage from the bladder.

**Acute Glaucoma.**

In this condition a large upward iridectomy should, if possible, be *immediately* performed. If postponement of the operation for some hours is unavoidable, every effort should be used to reduce the intra-ocular tension. Drops of eserine and cocaine should be instilled every hour, leeches should be applied to the temple, and fomentations to the eye, and the primæ viæ should be cleared out.

**Food and Sleep in Chorea.**

In severe cases of chorea food and sleep are sometimes hard to supply. Insomnia is rather an early than a late symptom and it may yield to strict quiet and a darkened room. But if sleep is absent over a period of twenty-four to thirty hours it must be procured by narcotics—chloral or bromide or the two together. Even the hypodermic injection of morphine is sometimes necessary.—*Dr. Sturges.*

